# Hybrid Benign Peripheral Nerve Sheath Tumors: A Comprehensive Literature Review with Emphasis on Their Clinical, Morphological and Genetic Features

**DOI:** 10.3390/diagnostics15070855

**Published:** 2025-03-27

**Authors:** Serena Salzano, Rosario Caltabiano, Magda Zanelli, Andrea Palicelli, Maurizio Zizzo, Nektarios Koufopoulos, Ioannis Boutas, Gaetano Magro, Valeria Barresi, Giuseppe Broggi

**Affiliations:** 1 Department of Medical and Surgical Sciences and Advanced Technologies “G.F. Ingrassia”, Anatomic Pathology, University of Catania, 95123 Catania, Italy; sere.salzano@gmail.com (S.S.); rosario.caltabiano@unict.it (R.C.); g.magro@unict.it (G.M.); giuseppe.broggi@unict.it (G.B.); 2 Pathology Unit, Azienda USL-IRCCS di Reggio Emilia, 42123 Reggio Emilia, Italy; andrea.palicelli@ausl.re.it; 3 Surgical Oncology Unit, Azienda USL-IRCCS di Reggio Emilia, 42123 Reggio Emilia, Italy; maurizio.zizzo@ausl.re.it; 4 Second Department of Pathology, Medical School, Attikon University Hospital, National and Kapodistrian University of Athens, 15772 Athens, Greece; nkoufo@med.uoa.gr (N.K.); iboutas@med.uoa.gr (I.B.); 5 Department of Diagnostics and Public Health, University of Verona, 37134 Verona, Italy; valeria.barresi@univr.it; 6 Pathology-Neuropathology Unit, Fondazione IRCCS Istituto Neurologico Carlo Besta, 20133 Milan, Italy

**Keywords:** hybrid benign peripheral nerve sheath tumors, CNS, schwannoma, neurofibroma, perineurioma

## Abstract

Hybrid Peripheral Nerve Sheath Tumors (HPNSTs) are rare benign neoplasms that exhibit a combination of histological features from multiple types of benign peripheral nerve sheath tumors, including schwannomas, neurofibromas, and perineuriomas. These tumors present a diagnostic challenge due to their morphological and histological variability. In this article, we aim to summarize the key morphological, histological, and molecular characteristics of HPNSTs, providing insights into their diagnostic approaches. We review the different hybrid subtypes, including schwannoma–perineurioma, schwannoma–neurofibroma, and perineurioma–neurofibroma, emphasizing their clinical features, genetic associations, and the role of surgical excision as the gold-standard treatment.

## 1. Introduction

Hybrid Peripheral Nerve Sheath Tumors (HPNSTs) are benign neoplasms that exhibit histological features of more than one type of peripheral nerve sheath tumor. The three types of peripheral nerve sheath tumors are schwannoma, neurofibroma, and perineurioma. Although recognized for some time, these tumors were only officially included in the World Health Organization (WHO) Classification of Tumors of Soft Tissue and Bone separately for the first time in the 4th edition and continued in the 5th edition of the WHO classification of CNS tumors, as well as soft tissue and bone tumors [1,2,3]. HPNSTs have been reported across all age groups, with a higher prevalence in young adults and no significant gender predilection. They exhibit a wide anatomical distribution, predominantly affecting somatic soft tissues, although cases involving bone have also been documented. A recent report by Chow et al. described an HPNST located in the femur [4]. The most common sites include the fingers (digits), especially in schwannoma/perineurioma hybrids, where they typically present as painless subcutaneous or dermal masses. Macroscopically, these tumors appear as well-circumscribed, firm, nodular lesions with a grayish cut surface, ranging from 1 to 8 cm in size [5]. In Table 1, all the main clinical and histological characteristics of the three types of PNSTs (schwannoma, neurofibroma, perineurioma) have been summarized.

Histologically, HPNSTs retain the morphologic and immunohistochemical features of their individual components, typically displaying a dual composition. Hybrid schwannoma/perineurioma tumors resemble perineuriomas in their lamellar, storiform, or whorled architecture, but their cellular morphology is predominantly schwannoma-like, with spindle-shaped cells, wavy nuclei, pale eosinophilic cytoplasm, and indistinct borders. Hybrid schwannoma/neurofibroma tumors, on the other hand, contain schwannoma-like Antoni A areas with nuclear palisading and Verocay bodies, alongside neurofibroma-like regions composed of fibroblasts, elongated wavy nuclei, and a collagenous or mucin-rich myxoid matrix arranged in an architecture that may be plexiform, albeit with a lack of consensus on diagnostic criteria [6]. The rare hybrid neurofibroma/perineurioma tumors present areas of perineuriomatous differentiation adjacent to plexiform neurofibroma-like regions [7]. Immunohistochemical (IHC) analysis confirmed these dual components: schwannoma/perineurioma hybrids were positive for S100 and SOX10 in schwannomatous regions, while perineuriomatous areas expressed EMA, Claudin-1, and GLUT-1. Schwannoma/neurofibroma hybrids exhibit strong S100 and SOX10 positivity in schwannomatous areas, whereas the neurofibroma component expresses S100, SOX10, EMA, and GLUT-1. In neurofibroma/perineurioma hybrids, the neurofibroma component follows the above-mentioned immunoprofile, while perineuriomatous components lack S100 expression [8]. HPNSTs are frequently associated with tumor syndromes, particularly NF2-related schwannomatosis, other schwannomatoses, and less frequently neurofibromatosis type 1 (NF1). More than 70% of patients with schwannomatoses develop single or multiple hybrid neurofibroma/schwannoma tumors, while over 25% of patients with NF2-related schwannomatosis exhibit at least one hybrid neurofibroma/schwannoma tumor. Hybrid neurofibroma/perineurioma tumors occur almost exclusively in NF1 [8]. Given their association with NF1, these tumors may undergo malignant transformation into malignant peripheral nerve sheath tumors (MPNSTs), although the precise recurrence and malignancy rates remain unclear. While rare, a few case reports describe local recurrence, and at least two cases of malignant transformation have been documented [9]. By analyzing the specific characteristics of this large group of tumors, this review aims to highlight the main diagnostic, prognostic, and therapeutic aspects, in order to improve the overall knowledge of and approach to these relatively uncommon entities.

## 2. Materials and Methods

### 2.1. Search Strategy

A literature review was conducted using PubMed/MEDLINE and Scopus to identify all English-language published cases of Hybrid Nerve Sheath Tumors in both adult and pediatric patients from 2014 to the present. The research utilized the following Medical Subject Headings (MeSH) terms: Hybrid Peripheral Nerve Sheath Tumor, Benign Neurofibroma/Schwannoma, Schwannoma/Perineurioma, and Neurofibroma/Perineurioma. Additionally, we manually reviewed the reference lists of relevant studies to identify articles that may have been missed during the electronic search.

### 2.2. Inclusion and Exclusion Criteria

The timeframe for the selected studies ranged from January 2014 to December 2024. Articles not addressing HPNSTs or those discussing them from a perspective other than their clinical, morphological, immunohistochemical, and genetic features were excluded.

Both case series (studies reporting at least two cases) and single case reports on this topic were included in the review, while comments, perspectives, guidelines, editorials, systematic reviews and/or meta-analyses, and manuscripts in languages other than English were excluded.

Papers available only as abstracts or those with text appearing too brief or non-informative were not included in the present review.

The clinical parameters analyzed included sex, age (median and range), anatomical location, median follow-up, HPNST type, genetic associations, tumor syndromes, histological and imaging features, local recurrence, and treatment (Table 2).

## 3. Results

**Figure 1:**
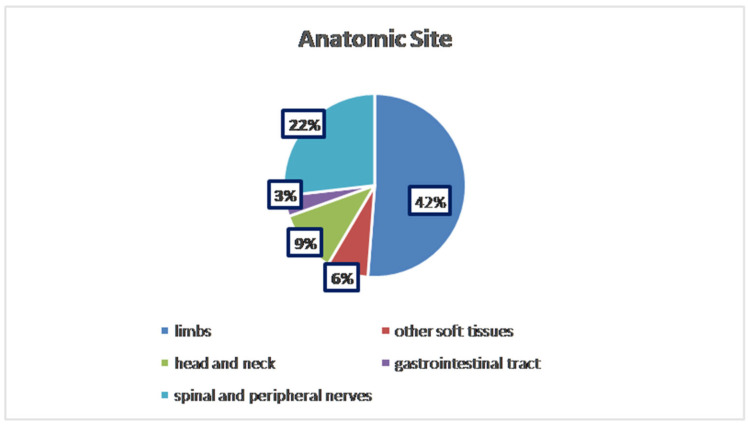
Distribution of anatomical sites.

To the best of our knowledge, about 112 cases of HPNSTs have been published to date. The analysis across 112 documented cases revealed a nearly equal distribution between genders, with 43% of patients being female (*n* = 48) and 37% male (*n* = 43). In 21 cases, the patients’ gender was not specified. The average age varied, but most cases fell within the 24–50 age range, with extremes spanning from 2 to 85 years old. Tumors were most commonly found in the limbs, accounting for 42% (47/112 cases), followed by spinal and peripheral nerves (22%; 25/112 cases), other soft tissue sites (retroperitoneum, vestibule, thigh, etc.) for 6% (8/112 cases), head and neck region (9%; 10/112 cases), and the gastrointestinal tract (3%; 4/112 cases). In the remaining 18 cases, the anatomical location of the lesion was not available (Figure 1).

**Figure 2:**
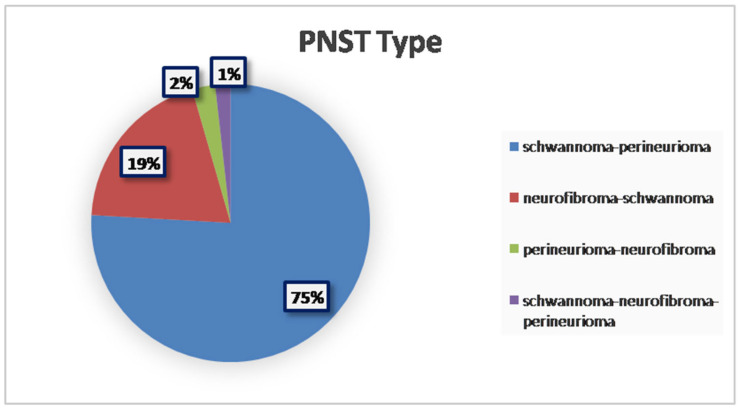
Prevalence of different PNST types.

From a histopathological perspective, the majority of tumors were classified as schwannoma–perineurioma hybrids, making up approximately three-quarters of the reported cases (75.89%; 85/112 cases). Other hybrid variants, including neurofibroma–schwannoma and perineurioma–neurofibroma, were less frequent (19.64%, 22/112 cases; 2.68%, 3/112 cases, respectively). To the best of our knowledge, only two reported cases described a hybrid tumor composed of three distinct components: schwannoma, neurofibroma, and perineurioma (1.79%; 2/112) [16] (Figure 2).

Regardless of histological subtype or anatomical location, all cases were treated surgically, reinforcing the fact that surgical excision with free margins remains the gold-standard treatment. The prognosis was highly favorable, with only one reported case of local recurrence, due to the presence of infiltrative margins and incomplete initial excision.

Genetic analysis was performed on 15% of the 112 cases. For a majority of these, no identifiable recognized genetic condition or related constitutional gene variant was found; however, as well as for the NF1 gene in neurofibromatosis, there are now known to be several genes in which mutations can underlie NF2 or schwannomatosis, including SMARCB1, LZTR1, or NF2 genes [27], and not all of the cases which had genetic analysis may have been tested for all relevant genes. However, a few instances exhibited somatic variants in genes such as BRAF, TERT, and NF2. Imaging findings varied based on tumor location, with MRI typically revealing well-circumscribed, lobulated masses. In cases affecting bone, osteolytic lesions were often present, while tumors in the gastrointestinal tract sometimes led to obstructive complications. In five cases, the patients were affected by neurofibromatosis type 1, and in fourteen cases, by schwannomatosis.

Furthermore, pertinent data were extracted and arranged in a narrative manner.

## 4. Discussion

### 4.1. Schwannoma–Perineurioma

**Figure 3:**
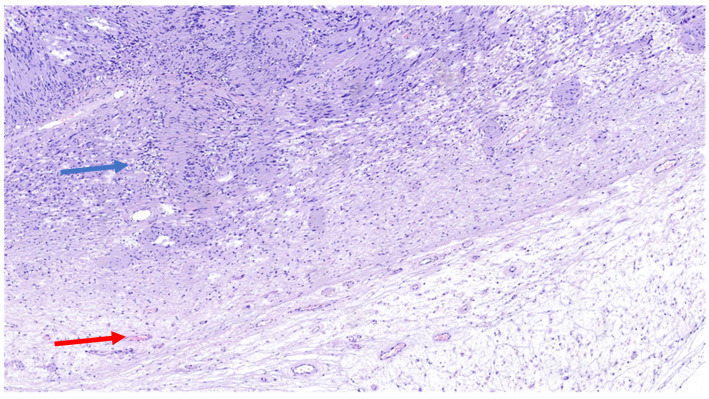
H&E (100× magnification) of a peripheral nerve sheath tumor with features of schwannoma and perineurioma. The schwannomatous component: areas of spindle-shaped cells arranged in bundles, with Verocay bodies (blue arrow), moderate cellularity. The perineurial component: uniform spindle cells arranged in a storiform pattern within a denser collagenous matrix (red arrow).

**Figure 4:**
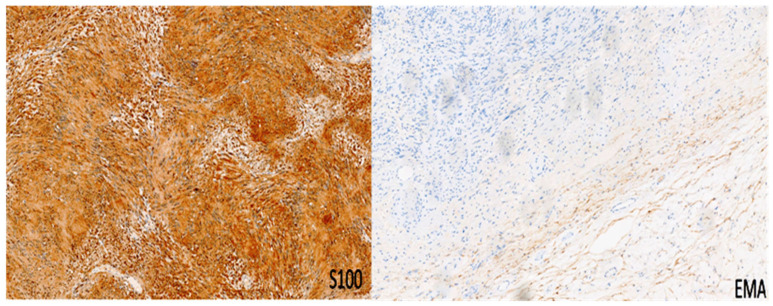
Immunohistochemistry for S100 and EMA in a peripheral nerve sheath tumor with features of schwannoma and perineurioma. S100 shows immunoreactivity in schwannomatous areas, while perineuriomatous components express EMA. Immunoperoxidase; original magnifications: 100×.

The schwannoma–perineurioma hybrid is the most reported subtype and typically occurs sporadically. Macroscopically, these tumors are well circumscribed but unencapsulated and are composed of spindle cells with wavy, tapering nuclei, eosinophilic cytoplasm, and indistinct cell borders. Histologically, they display a perineurioma-like pattern, with storiform, lamellar, or whorled growth, while still maintaining a schwannoma-like cytomorphology (Figure 3). Degenerative changes similar to those seen in ancient schwannomas, such as myxoid degeneration and cystic alterations, may also be present. Immunohistochemically, these tumors exhibit S100 and SOX10 immunoreactivity in schwannomatous areas, while perineuriomatous components express EMA, Claudin-1, and GLUT-1 (Figure 4). Unlike schwannoma–neurofibroma hybrids, these tumors are usually sporadic and not strongly associated with genetic syndromes. They are considered benign with an extremely low recurrence risk, although rare cases of low-grade malignant potential have been reported in the literature [5]. It has also been reported that the majority of hybrid schwannoma–perineuriomas harbor VGLL3 rearrangements [28,29].

### 4.2. Schwannoma–Neurofibroma

**Figure 5:**
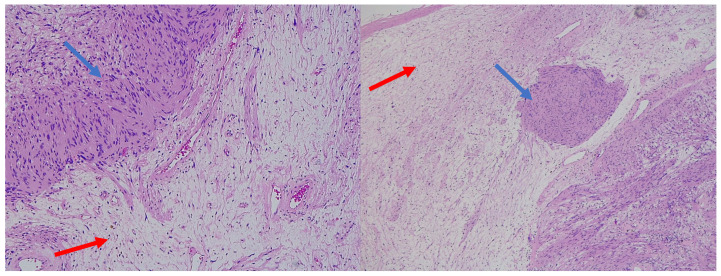
H&E (100× magnification) of a peripheral nerve sheath tumor with features of schwannoma and neurofibroma. The schwannomatous component: elongated cellular bundles, Verocay bodies (blue arrow), and an Antoni A/B pattern. The neurofibromatous component: heterogeneous cellular population, scattered spindle cells within a more myxoid matrix (red arrow).

**Figure 6:**
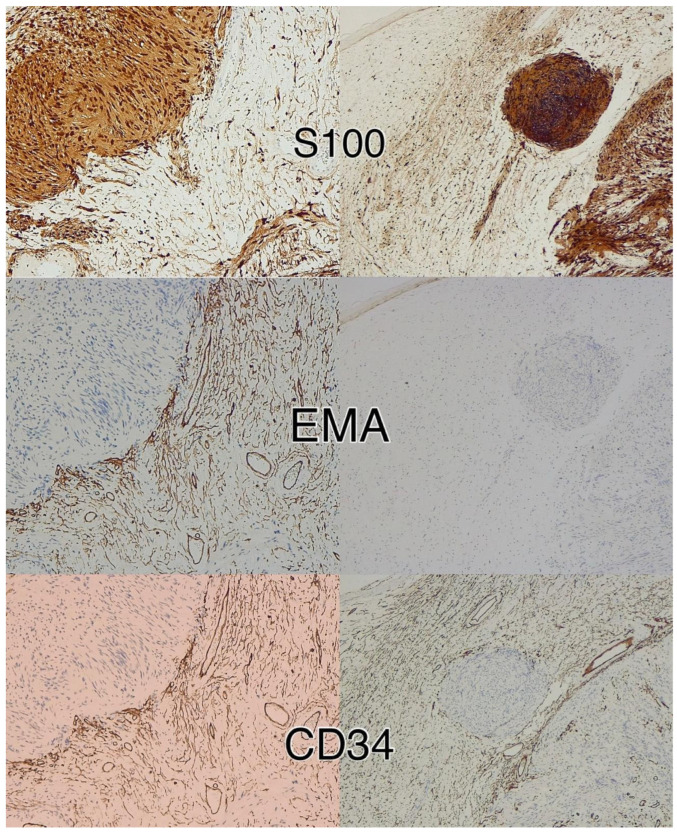
Immunohistochemistry for S100, CD34, and EMA in a peripheral nerve sheath tumor with features of schwannoma and neurofibroma. Schwannomatous areas are strongly positive for S100, whereas the neurofibroma component expresses S100, CD34, and EMA. Immunoperoxidase; original magnifications 100×.

The schwannoma–neurofibroma hybrid is one of the more commonly described HPNST variants and is frequently associated with NF1- and NF2-related schwannomatosis, as well as other forms of schwannomatosis. Although accurate diagnostic criteria have not yet been established [6], these tumors exhibit distinct cellular components: Schwann-cell nodules exhibiting Antoni A areas with nuclear palisading and Verocay bodies, and strong immunoreactivity for SOX10 and S100, while intermixed neurofibroma-like regions consist of elongated, wavy nuclei, fibroblasts, and a collagen-rich myxoid matrix, often organized in a plexiform arrangement (Figure 5), with scattered S100/SOX10 staining, and entrapped NFP-positive axons. As mentioned above, immunohistochemically, schwannomatous areas are strongly positive for S100 and SOX10, whereas the neurofibroma component expresses S100, SOX10, CD34, EMA, and GLUT-1 (Figure 6). These tumors are usually slow-growing and asymptomatic, and they have a low recurrence rate following surgical excision. However, when associated with NF1, there is a potential risk of malignant transformation into MPNSTs [7].

### 4.3. Perineurioma–Neurofibroma

The perineurioma–neurofibroma hybrid is the rarest subtype of HPNSTs and is almost always associated with NF1. These tumors contain both perineuriomatous and neurofibromatous regions, with the latter frequently exhibiting a plexiform architecture. The perineurioma component consists of spindle cells with elongated nuclei, arranged in thin fascicles or whorled patterns, whereas the neurofibroma component comprises fibroblasts, Schwann cells, and an extracellular matrix rich in mucin and collagen fibers. Immunohistochemically, the neurofibroma component is positive for S100, SOX10, EMA, Claudin-1, and GLUT-1, while the perineurioma component does not express S100. This subtype has been reported less frequently than other hybrid PNSTs, and its clinical behavior is not well characterized. However, given its strong association with NF1, there is a potential risk of malignant transformation, similar to other NF1-associated tumors [7]. Due to their rarity, their classification is still to be defined, and it is not clear whether they represent a “true” distinct tumor category or simply neurofibromas rich in perineurial cells.

## 5. Conclusions

HPNSTs are a rare and complex group of neoplasms that primarily affect young to middle-aged adults and have no significant gender predisposition. They most commonly occur in the limbs and soft tissues and are predominantly composed of schwannoma–perineurioma hybrids. Surgical excision remains the gold standard for treatment, with a favorable prognosis and an extremely low recurrence rate. Despite the lack of consistent genetic associations, some cases exhibit somatic mutations in genes such as BRAF, TERT, and NF2, suggesting potential molecular underpinnings of these tumors. The association with genetic syndromes, particularly NF1, warrants careful surveillance, as there is a possibility of malignant transformation, although this outcome remains unusual. Our findings align with those of Hornick et al. [13], who reported a predominance of schwannoma–perineurioma hybrids and an extremely low rate of local recurrence (probably due to infiltrative margins on histology and a lack of complete surgical resection). It must be mentioned that rare cases of benign cutaneous plexiform hybrid tumors of perineurioma and cellular neurothekeoma (BCPHTPCNs) have been recently described [30,31,32]; these lesions tend to arise as solitary papules in the perioral area [16,28,29]. BCPHTPCNs exhibit combined morphologic features of both perineurioma and cellular neurothekeoma, frequently arranged in a plexiform pattern [30,31,32]. Neurothekeomas are rare, benign soft tissue tumors that typically appear on the head and neck. The histological patterns of neurothekeomas can vary, including myxoid, cellular, or mixed types, with the variation primarily dependent on the amount of myxoid matrix present [33]. However, as neurothekeomas are not properly considered peripheral nerve sheath tumors, we excluded this hybrid form from the present review.

Finally, further research into the molecular characteristics (e.g., *NF2*, *BRAF*, and *TERT* mutations) of these tumors may offer greater insight into their pathogenesis and potential therapeutic targets. Overall, the current data support a highly favorable outcome for most patients, particularly when treated surgically, although long-term monitoring is essential for those with genetic predispositions.

## Figures and Tables

**Table 1:** Classic and distinguishing features of each of the 3 main types of PNSTs.

	Epidemiology	Sites	Histopathology	IHC	Genetic Associations	Tumor Syndromes
**Schwannoma**	All ages; M = F	Limbs	Biphasic: compact hypercellular Antoni A areas and myxoid hypocellular Antoni B areas; nuclear palisading around fibrillary process (Verocay bodies).	S100 + SOX10 + Podoplanin + EMA-	SMARCB1 NF2	Neurofibromatosis type 2, schwannomatosis, Carney complex
**Neurofibroma**	Second–third decades; M = F	Body surface usually in the head and neck region	Schwann cells with wire-like collagen fibrils, stromal mucosubstances, mast cells, Wagner–Meissner corpuscles, Pacinian corpuscles, axons, fibroblasts, and collagen.	S100 + CD34 + SOX10 + Factor XIIIa + Calretinin + EMA + Podoplanin +	NF1	Neurofibromatosis type 1
**Perineurioma**	Young adults; M = F	Limbs	Multiple small “onion bulbs” expanding the affected nerve, consisting of concentric layers of perineurial cells ensheathing a central axon and Schwann cell	EMA + CD34 + S100 + GFAP – CD57 −	TRAF7 NF1 NF2	No

**Table 2:** Cases of HPNSTs reported in the literature to date.

Author	Cases (*n*)	Gender	Age	Anatomic Site	PNST Type	Follow-Up	Histopathology	Genetic Associations	Tumor Syndromes	Imaging	Local Recurrence	Treatment
Agaimy [10]	2	1 female 1 male	50; 17	gastric antrum; vermiform appendix	Schwannoma–perineurioma	8–12 months	storiform, lamellar, fascicular patterns	No	No	N.A.	No	surgery
Bergamini [11]	1	Female	54	mandibular body	Schwannoma–perineurioma	2 years	spindle to epithelioid cells; wavy and hyperchromatic nuclei.	No	No	N.A.	No	surgery
Chow [4]	1	Male	18	femur	Schwannoma–perineurioma	7 months	storiform pattern; wavy nuclei	No	No	Plain radiograph: displaced fracture in right femur diaphysis with expansile osteolytic lesion with well-defined borders.	No	surgery
Lang [12]	1	Female	28	tibia	Schwannoma–perineurioma	8 months	bland-looking spindle to epithelioid cells	No	No	Radiological examination revealed an oval eccentric osteolytic lesion in the proximal tibia.	No	surgery
Hornick [13]	42	22 female 20 male	mean age: 38 y; range: 2 to 85	lower limb (19), upper limb (12), head and neck (6), trunk (4), colon (1)	Schwannoma–perineurioma	24 months	storiform, whorled/lamellar pattern. Infiltrative margins in 1 case	No	No	N.A.	1/42	surgery
Singh [14]	24	15 Female 9 Male	mean age: 50 years (range: 24–78 years)	spinal roots (19) intramuscular (5)	Schwannoma--perineurioma	N.A.	dual Schwannian and perineural differentiation.	No	No	MRI: lobular contour, ancient changes, fascicular sign, entering nerve sign, exiting nerve sign, target sign, and split fat sign	No	surgery
Ud Din [15]	5	1 female 4 male	mean: 24 years; median: 12 years	big toe of left foot (1), soft tissue of left thigh (1), soft tissue of right thigh (1), soft tissue of neck region (1), retroperitoneum (1)	Schwannoma–perineurioma (3), neurofibroma–perineurioma (1), schwannoma–neurofibroma (1)	N.A.	Spindle cells	No	No	N.A.	No	surgery
Michal [3]	6	5 Female 1 Male	mean age: 33 years	fingers (5), thenar eminence of the hand (1)	Schwannoma–retiform perineurioma	N.A.	myxoid and pseudocystic changes.	No	No	N.A.	No	surgery
Kuroda [16]	1	Male	58	middle meatus of the nose	Schwannoma–neurofibroma–perineurioma	N.A.	schwannoma, neurofibroma, and perineurioma differentiation.	No	No	N.A.	No	surgery
Chijiiwa [17]	1	Male	41	right thigh	Schwannoma–neurofibroma	N.A.	dual histology (schwannoma and neurofibroma).	No	No	Well-circumscribed intramuscular mass with low-to-intermediate signal intensity on T1-weighted sequences and higher signal intensity peripherally and lower signal intensity centrally	No	surgery
Leite [18]	1	Female	68	lower vestibule	Neurofibroma–schwannoma	N.A.	dual histology (schwannoma and neurofibroma).	No	No	N.A.	No	surgery
Emanuel [19].	1	Male	48	colon	Perineurioma–schwannoma	N.A.	whorling or storiform growth pattern mixed with spindle wavy cells	No	No	Colonoscopy and computed tomography scan revealed an obstructing colonic mass, causing intussusception and pneumatosis of the descending/upper sigmoid colon and necessitating an emergency left hemicolectomy	No	surgery
Colazo [20]	1	Male	74	ulnar nerve	Neurofibroma–schwannoma	N.A.	“thumbprint” pattern of neurofibroma; spindled cells arranged in fascicles and palisades	BRAF, TERT and NF2	No	suspicious for a PNST, but an ultrasound-guided biopsy was equivocal; mimicked glandular schwannoma	No	surgery
Goyal-Honavar [21]	1	Female	22	trigeminal nerve	Schwannoma–perineurioma.	5 months	biphasic pattern with areas of spindle-shaped cells	No	No	MRI: solid and cystic extra-axial tumor in the right cerebellopontine angle cistern	No	surgery
Hong [22]	1	Male	54	orbit	Neurofibroma–schwannoma	4 months	spindle cells	No	No	MRI: well-demarcated tumor of 43 mm compressing the optic nerve medially	No	surgery
Kacerovska [8]	4	N.A.	N.A.	N.A.	Schwannian–perineurioma (3) neurofibroma–perineurioma (1)	N.A.	spindle cells	NF1	Type 1 neurofibromatosis	N.A.	No	surgery
Mitsui [23]	3	N.A.	N.A.	upper back, forearm, and thigh	Schwannoma–neurofibroma	N.A.	spindle cells	No	No	N.A.	No	surgery
Yusuke Inatomi [24]	1	Male	30	head	Perineurioma–neurofibroma	2 months	N.A.	NF1	Type 1 neurofibromatosis	N.A.	No	surgery
Harder [25]	14	N.A.	N.A.	N.A.	Neurofibroma–schwannoma	N.A.	N.A.	NF2	Schwannomatosis	N.A.	No	surgery
McLaughlin [26]	1	Male	30	upper thoracic–region	Schwannoma–perineurioma–neurofibroma	5 years	schwannoma, neurofibroma, and perineurioma differentiation.	NF1	Type 1 neurofibromatosis	N.A.	No	surgery

N.A.: not available.

## Data Availability

No new data were created or analyzed in this study.

## Abbreviations

The following abbreviations are used in this manuscript:

HPNSTs	Hybrid Peripheral Nerve Sheath Tumors
WHO	World Health Organization
PNSTs	Peripheral nerve sheath tumors
MPNSTs	Malignant peripheral nerve sheath tumors
MeSH	Medical Subject Headings
IHC	Immunohistochemical
NF1	Neurofibromatosis type 1
BCPHTPCN	Benign cutaneous plexiform hybrid tumor of perineurioma and cellular neurothekeoma
